# Amelioration of behavioral and histological impairments in somatosensory cortex injury rats by limbal mesenchymal stem cell transplantation

**DOI:** 10.1515/tnsci-2022-0346

**Published:** 2024-08-14

**Authors:** Ali Derakhshani, Farahnaz Taheri, Nima Geraminia, Lily Mohammadipoor-ghasemabad, Mansoureh Sabzalizadeh, Farzaneh Vafee, Mohammad Reza Afarinesh, Vahid Sheibani

**Affiliations:** Hydatid Disease Research Center, Kerman University of Medical Sciences, Kerman, Iran; Neurology Research Center, Institute of Neuropharmacology, Kerman University of Medical Sciences, Kerman, Iran; Kerman Neuroscience Research Center, Institute of Neuropharmachology, Kerman University of Medical Sciences, P. O. Box: 76198-13159, Kerman, Iran; Cognitive Neuroscience Research Center, Institute of Neuropharmachology, Kerman University of Medical Sciences, Kerman, Iran; Neuroscience Research Center, Mashhad University of Medical Sciences, Mashhad, Iran

**Keywords:** cold lesion, limbal mesenchymal stem cell, somatosensory cortex, cognitive impairment

## Abstract

**Introduction:**

Cortical lesions can cause major sensory and motor impairments, representing a significant challenge in neuroscience and clinical medicine. Limbal mesenchymal stem cells (LMSCs), renowned for their remarkable ability to proliferate and distinct characteristics within the corneal epithelium, offer a promising opportunity for regenerative treatments. This study aimed to assess whether the transplantation of LMSCs could improve tactile ability in rats with lesions of the barrel cortex.

**Methods:**

In this experimental study, we divided 21 rats into three groups: a control group, a lesion group with cortical cold lesion induction but no stem cell treatment, and a group receiving LMSC transplantation following cold lesion induction. We conducted 3-week sensory assessments using a texture discrimination test and an open-field test. We also performed Nissl staining to assess changes on the cellular level.

**Results:**

Rats in the LMSC transplantation group demonstrated significant improvements in their ability to discrimination textures during the second and third weeks compared to those in the lesion group. The open-field test results showed an increased exploratory behavior of rats in the LMSC transplantation group by the third week compared to the lesion group. Additionally, Nissl staining revealed cellular alterations in the damaged cortex, with a significant distinction observed between rats in the LMSCs and lesion group.

**Conclusion:**

The findings suggest that LMSC transplantation enhances sensory recovery in rats with cortical lesions, particularly their ability to discriminate textures. LMSC transplantation benefits brain tissue reparation after a cold lesion on the somatosensory cortex.

## 1 Introduction

Cortex injury can occur as a result of various head impacts, including those from car accidents, sports-related incidents, and falls. It often causes significant and enduring impairments in cognitive, sensorimotor, social, emotional, and behavioral functions [1]. Numerous molecular cascades are triggered to induce secondary brain damage in both trauma and cerebral ischemic stroke [2]. Comparable processes, such as oxidative stress, excitotoxicity, inflammation, and apoptosis, collectively contribute to cell and tissue integrity deterioration, resulting in sustained alterations in axon pathology from minutes to weeks [3]. Traumatic brain injury (TBI) is a complex disease process involving primary and secondary injury mechanisms [4]. Primary injury occurs from the mechanical disruption of brain tissue [5] while Subsequently, secondary injury occurs over time, leading to cell death, tissue damage, and atrophy [6]. Rodents use their prominent facial whiskers, known as vibrissae, actively during object exploration. These vibrissae are linked to the barrel cortex through a complex network of sensorimotor connections. This network is crucial in regulating various behaviors that are dependent on whisker usage [7]. Rodents actively sweep their whiskers across surfaces [8]. Through clearly elucidated sensory pathways, the sensory impulses originating from the whiskers on a rat’s face are translated into signals that activate cells within the somatosensory cortical area called the whisker barrel field cortex (BFC). The BFC comprises interhemispheric connections, and a hypothesis suggests that these connections act as a pathway for signaling the existence of a lesion. Emphasizing this aspect holds considerable significance. Based on connectional studies, it has been observed that the left and right BFC exhibit an intricate arrangement of commissural projections. These projections are believed to facilitate the bilateral responses evoked by whisker stimulation [9,10]. In rodent models, injury or stroke induced in the parietal lobe results in sensory and motor deficits, causing tactile dysfunction [11]. Following global and focal ischemia, abnormalities have been documented in the somatosensory circuit’s ability to respond to metabolically physiological activation [12].

An innovative approach to addressing neurodegenerative disorders within the central nervous system involves stem cell-based therapy. This strategy holds promise in targeting the fundamental pathophysiology due to the inherent abilities of stem cells. These cells can reduce inflammation, restore the integrity of the blood-brain barrier, and stimulate neural regeneration by releasing specific neurotrophic factors [13]. Limbal mesenchymal stem cells (LMSCs) represent an undifferentiated cell population with notable proliferative capabilities, making them well-suited to regenerate and restore corneal tissue [14]. LMSCs do not reveal the expression of markers specific to differentiated and mature corneal epithelium [14]. This unique set of attributes has driven researchers to dedicate considerable time over the years in search of an authentic marker for LMSCs that could facilitate the potential separation of these cells for therapeutic applications. Because of its multifaceted roles encompassing light transmission, refraction, and protection of underlying ocular structures against environmental hazards, the cornea plays a pivotal role in facilitating normal vision [15]. Distinguished by its remarkable regenerative capacity and rapid ocular surface repair abilities, the corneal epithelium is recognized for its propensity to proliferate and migrate centripetally at the border between the cornea and the sclera, also known as the limbus [16]. Nevertheless, it is still unclear how LMSC growth and neurotrophic factors functionally assist central nervous system neurons. The neurotrophic and neuroprotective characteristics of stromal stem cells derived from the human limbus were investigated in a study. According to Liang et al., soluble paracrine substances secreted by LMSCs enhance neurite outgrowth and shield cortical neurons from hypoxic insults, ultimately leading to improved functional recovery following ischemic stroke [17]. Our study aimed to investigate the potential therapeutic benefits of LMSC transplantation following cortical lesions, mainly focusing on its impact on tactile discrimination abilities. Through a series of behavioral tests and histological analysis, we sought to elucidate the role of LMSCs in enhancing sensory recovery in an animal model of post-injury neurological function.

## 2 Methods

### 2.1 Animals

This experimental study obtained approval from the research ethics committee of the Kerman neuroscience research renter (KNRC) under the ethics (IR.KMU.AEC.1402.060). In addition, for investigations involving human limbal tissue, informed consent has been obtained from the participants involved. Twenty-one male *Wistar* rats, weighing between 200 and 230 g, were included in the study. These rats were housed in sanitary cages within a controlled environment. The temperature was regulated, and ventilation was maintained while a steady 12-h light/dark cycle was upheld. Furthermore, the rats were provided unrestricted access to a standard diet and water supply.

### 2.2 Cell isolation and culture

**Figure 1:**
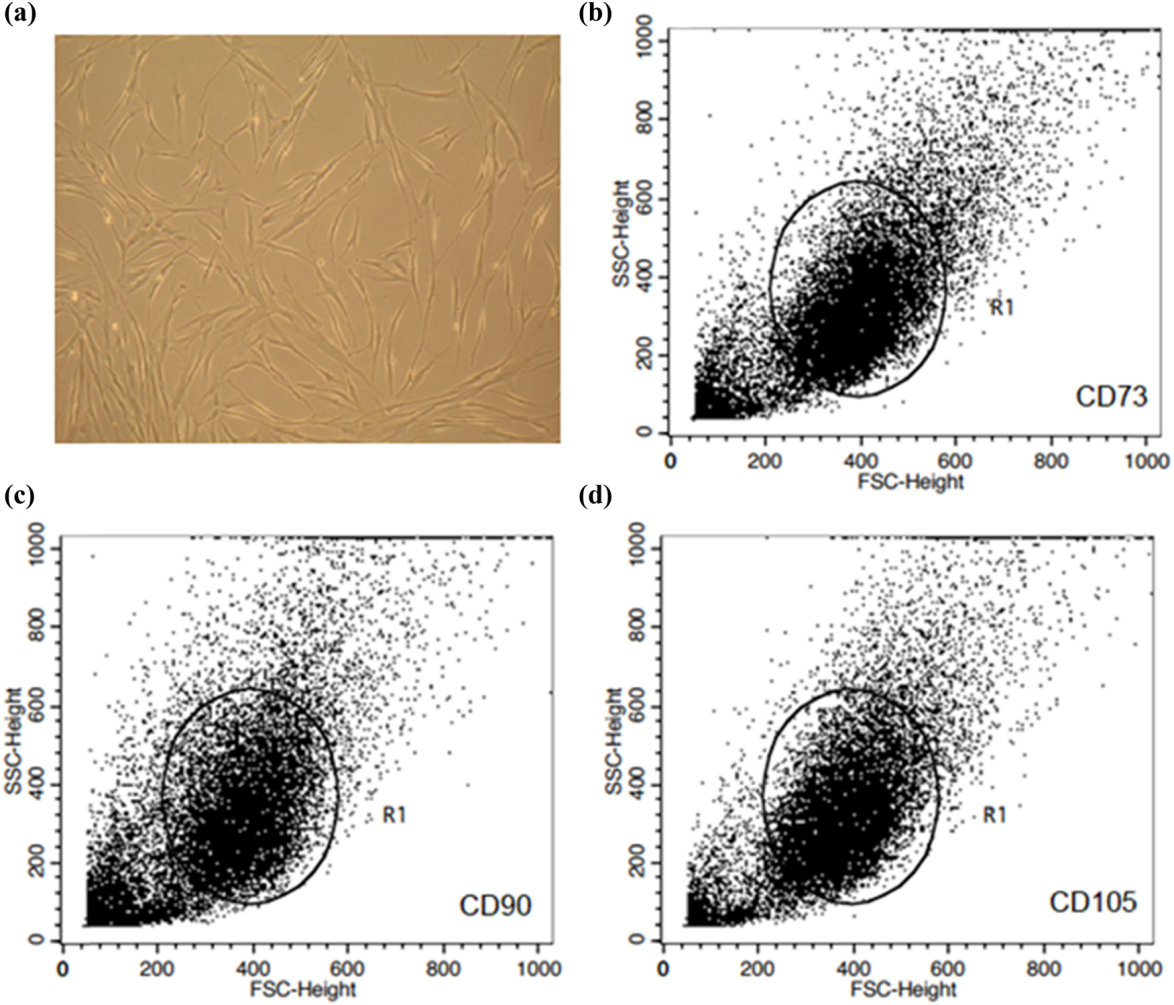
LMSCs morphology. Within 8 to 14 days after the isolation process, LMSCs appeared as adherent cells on the bottom of culture dishes. The isolation success rate was 70 and 80% for LMSCs. The cell morphology of cultured LMSCs was confirmed using phase contrast microscopy. Homogenous LMSCs showed morphologic features similar to fibroblasts (a). Scale bar = 20 μm. All the surface markers tested were similarly expressed on LMSCs. the populations of LMSCs were positive for mesenchymal markers CD105, CD90, and CD73 (b–d).

Human MSCs were isolated from limbal tissue from 5 different donors. The tissue was minced using two scalpels and disintegrated as small as possible for cell isolation. The minced tissue was then digested at 37°C with NB6 collagenase (1 mg/mL; *Nordmark*, Germany). Cells were then separated and centrifugation at 1,200× rpm. The cells were plated for initial cell culture and cultured at 37°C in an atmosphere of 5% CO_2_ in humid air. Dulbecco’s modified Eagle’s medium (DMEM; Sigma, Germany) was used with a physiologic glucose concentration (100 mg/dL) supplemented with 10% fetal bovine serum (FBS; Biochrom, Berlin, Germany) as the standard culture medium. Primary isolated cells were intensively washed with PBS after 18–24 h of initial plating to remove debris and non-adherent cells. The medium was then replaced every three days. Sub confluent cells were passaged by trypsinization (Figure 1a).

### 2.3 Cell characterizations using flow cytometry

Cell morphology was examined by phase contrast microscopy. Flow cytometric analysis was used to show the characteristic marker expression of cultured LMSCs. Cells were detached from the cell culture flask and stained with directly labeled antibodies CD73 (1:20, BD-555824, BD-biosciences, USA), CD90 (1:5, BD-555596, BD-biosciences, USA), and CD105 antibodies (1:5, BD-561443, BD-biosciences, USA). The labeled LMSCs were then measured using a flow cytometer (FACS Calibur model, BD-Bioscience- USA). All experiments included negative controls with corresponding isotype controls. Data analysis was performed using Cellquest Pro v5.1 software. The immunophenotypic characteristics of the cells are depicted in Figure 1b–d.

### 2.4 Surgery and transplantation of stem cells

**Figure 2:**
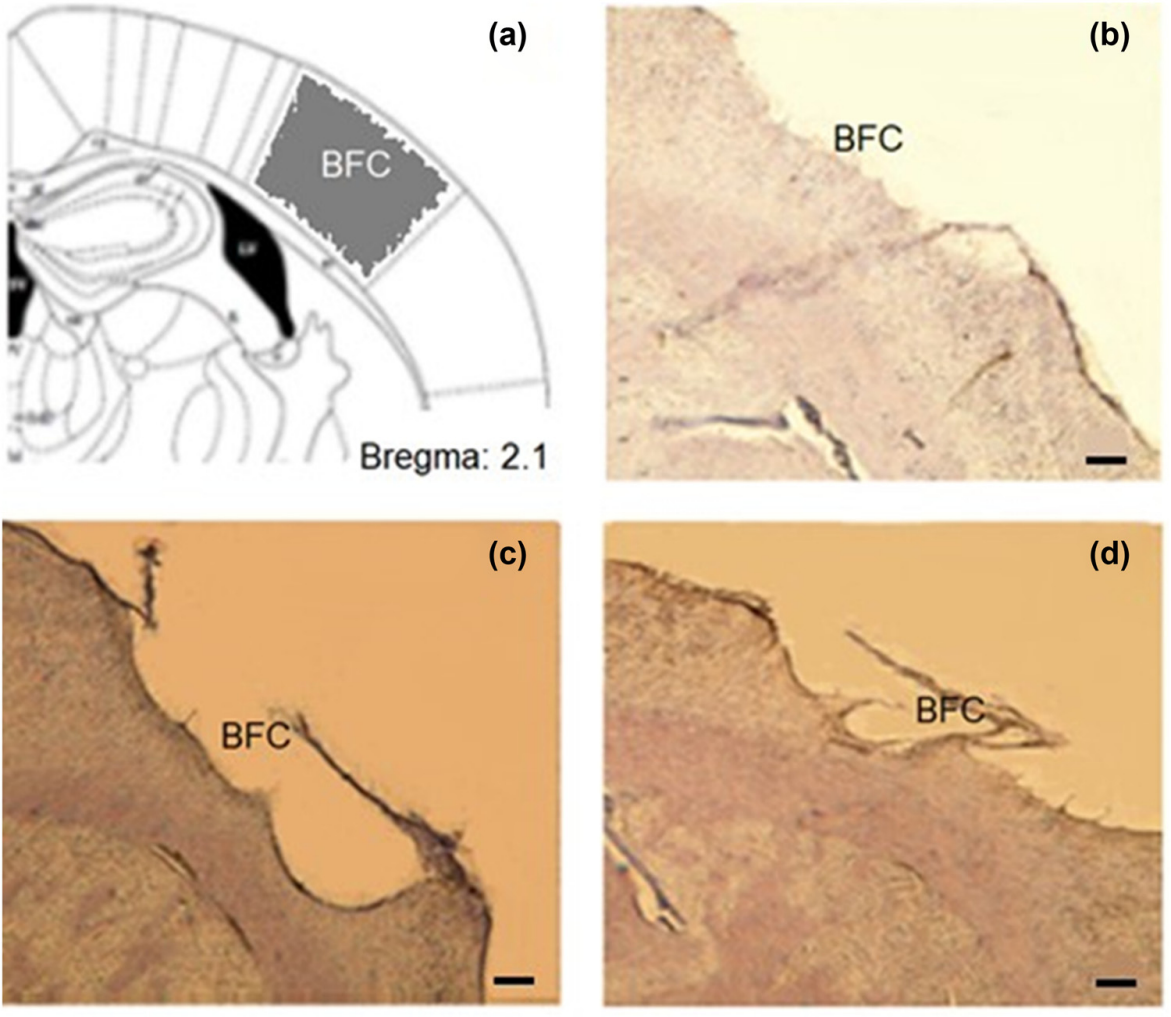
Transcranial Cryogenic Lesion: The panel (a) shows a schematic coronal section of the rat barrel field (BFC) of cortex [15]. Panels (b)–(d) show the Nissl staining of 3 coronal sections belonging to 3 rats in the cold lesion group. Scale bar = 10 μm.

**Figure 3:**
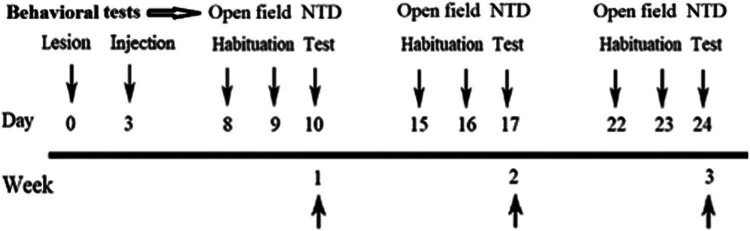
Experimental timeline. On day 0, a cold lesion is induced in the right barrel cortex area. Three days after lesioning, LMSCs were injected into the lesioned area. The behavioral test was performed for three consecutive weeks post-injection. Each test followed two sessions of habituation. NTD: Novel texture discrimination.

The rats underwent anesthesia through an intraperitoneal injection of ketamine (100 mg/kg) and xylazine (10 mg/kg) before being positioned within a stereotaxic apparatus (Stolting, USA). A dental drill was employed to remove the skull covering the right barrel cortical area carefully. Additionally, a scalpel was utilized to make an incision in the skin over the parietal cortex. The barrel cortex was localized using a combination of methods, including stereotaxic coordinates (4–7 mm lateral to the midline and 1–4 mm posterior to the bregma), as well as reference to vascular landmarks [13]. Transcranial cryogenic lesions were induced in the barrel cortex. To summarize, A 3 mm diameter copper cylinder, cooled with liquid nitrogen, was placed for 80 s on the exposed calvarium directly above the somatosensory cortex. Lesions were created unilaterally (Figure 2). Following this procedure, the animal’s incision was sutured, and it was returned to its cage [18]. Following the surgical procedures, rats were initially housed separately for three days. On the third day after the stem cell injection, the rats were placed in the cages in pairs (Figure 3). The three experimental groups were distributed randomly among the rats. The control group (*n* = 7) remained unchanged. 80 μl of Dulbecco’s Modified Eagle Medium (DMEM) were directly injected into the lesion site after the third day for the lesion group (*n* = 7). On the third day after the creation of the lesion, a total of 1 × 10^6^ undifferentiated [13] limbal stem cells (LMSCs) were injected into the lesion site for the stem cells group (*n* = 7). In total, 21 male rats were used in our study.

### 2.5 Behavior assessments

Our study aimed to investigate the impact of LMSCs transplantation on the recovery of sensory abilities in a barrel cortex with a cold lesion. The experiments involved three primary experimental groups, each contributing uniquely to our research.

#### 2.5.1 Open-field test

Rats’ whiskers are multi-functional sensory organs, crucial for tasks ranging from object detection, localization, shape, and texture assessment to discrimination and even movement coordination, including distance detection and motor coordination [19]. As rodents heavily depend on their whiskers for exploring new environments during their movements, we initiated our investigation with an open-field test to evaluate their inherent spatial exploration abilities. At the start of the test, the rats were placed into the central area of a chamber measuring 50 × 50 × 40 cm. Over a duration of 5 min, their movements were monitored and recorded using a video camera [20,21].

#### 2.5.2 Novel texture discrimination

The assay relies on the animals’ inherent inclination to explore novel objects, making it feasible to be performed after a period of habituation and without the need for extensive training. Before each testing session, the rats underwent two habituation sessions within the testing box. This procedure was implemented to enhance their exploratory activity during the subsequent tests. During the experimental phase, rats were situated in a plexiglass box measuring 50 × 50 × 40 cm for 10 min each day over two consecutive days leading up to the test day. On the test day, each rat was placed inside the box to explore two identical textures for 5 min, and then the rat was returned to its home cage. The box was thoroughly cleaned with 70% ethanol, and one of the previously presented textures was substituted with a novel texture. After a 5-min interval, the rat was again introduced into the box and granted 5 min to explore the textures, marking the test phase. These textures were plates measuring 6 × 15 cm, each covered with either rough (P120) or smooth (P220) sandpaper [22]. Notably, the behavioral test was performed under video camera surveillance in a quiet room with dim red lighting (40 W) above the box. To prevent the repeated use of the same objects throughout the experiment, three identical plates were employed for each grade of sandpaper. For the analysis, data from rats that did not explore both objects during the learning or test phase were excluded. The Preference Index (PI) of the test phase was computed as the ratio of time spent exploring the novel object to the total exploration time, encompassing the time spent exploring both the new and the old object [13,23].

### 2.6 Histological evaluation

Following the completion of behavioral tests, a subset of subjects (comprising 3 rats per group) underwent deep anesthesia using a combination of ketamine (80 mg/kg) and xylazine (10 mg/kg). Coronal sections of the brain were then prepared, each with a thickness of 7 μm, and subsequently stained using Nissl staining [24].

### 2.7 Statistical analysis

All statistical analyses were conducted using SPSS software. Behavioral data is presented as the mean ± SEM (Standard Error of the Mean). A significance level of 0.05 (*P* < 0.05) was deemed statistically significant. Before data analysis, missing data were imputed by Expectation Maximum method. Repeated measurement analysis and one-way ANOVA were applied to address the presence of interaction between time and group, as well as their main effects. Histology data were analyzed using a one-way ANOVA test followed by Bonferroni *post hoc*.


**Ethical approval:** The research related to animals’ use has been complied with all the relevant national regulations and institutional policies for the care and use of animals. This experimental study obtained approval from the research ethics committee of the Kerman neuroscience research renter (KNRC) under the ethics (IR.KMU.AEC.1402.060).

## 3 Results

### 3.1 Open field test

In each test, we measured the total distance moved by the rats as they explored the open field maze. Since a repeated measurement showed that there was not any significancy for time (F2, 36 = 0.97, *P* = 0.38) and also interaction of treatment group*time (F4, 36 = 0.4, *P* = 0.77), a one-way ANOVA was conducted for group variable to study a main effect change. During the first week (week 1), the group with lesions showed no significant statistical difference in total distance moved compared to the control group. However, there were no significant differences between the lesion group and the LMSCs group. In the subsequent test at week 2, the results were similar to the first week, with no significant differences between the LMSCs group and the lesion group. The lesion group had a lower distance moved than the control group, but this difference was not significant.

**Figure 4:**
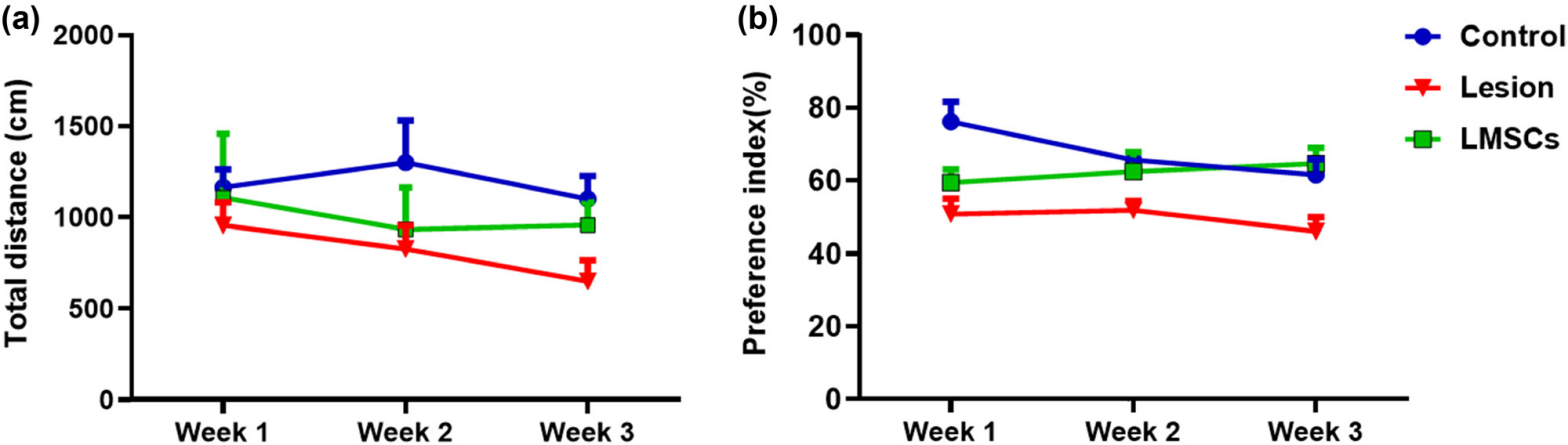
Effects of limbal stem cell transplantation (LMSCs) on the open field test (a) and texture discrimination test (b) in different weeks. In the open field test, the lesion group showed deficits in exploratory behavior, as evidenced by a reduced total distance moved, compared to the control group. The texture discrimination test was conducted weekly after the LMSCs transplantation. Importantly, in week 3, the PI for the new texture showed a significant increase in the group that received LMSCs transplantation compared to the group that received the lesion The data are presented as mean ± SEM. *n* = 7. *P < 0.05 vs Control and ^##^ *P* < 0.01 vs Lesion.

By the third test (week 3), (F2, 18 = 3.96, *P* = 0.03) there was a significant decrease in the total distance moved between the control group and the lesion group (*P* < 0.05). The performance of the LMSCs group improved, but there was no significant difference between the LMSCs group and the lesion group (Figure 4a).

### 3.2 Novel texture discrimination test (NTD)

During the NTD test, we evaluated the rats’ preference for the new texture by calculating the PI, which indicates the ratio of time spent exploring the new object compared to the total exploration time. Since a repeated measurement showed that there was not any significancy for time (F2, 36 = 1.05, *P* = 0.36) and also interaction of treatment group*time (F4, 36 = 1.6, *P* = 0.25), a one-way ANOVA was conducted for group variable to study a main effect change. Further analysis revealed that during the initial week, (F2, 18 = 8.12, *P* = 0.003) the lesion group displayed a significant difference in PI compared to the control group (*P* < 0.01). However, there was no significant difference in PI between the lesion and LMSCs groups. In the second week, (F2, 18 = 3.91, *P* = 0.03) the lesion group displayed a significant decrease in PI compared to the control (*P* < 0.05), In contrast, the LMSCs group’s performance improved to the point where there was no significant difference between the performance of LMSCs and control groups. Furthermore, there was no significant difference in PI between the LMSCs and lesion groups. In the third week, (F2, 18 = 7.14, *P* = 0.005) the lesion’s performance remained significantly lower than the control’s (*P* < 0.05) while a significant increase was observed in PI in the LMSCs compared to the lesion groups (*P* < 0.01) (Figure 4b).

### 3.3 Histology evaluations

**Figure 5:**
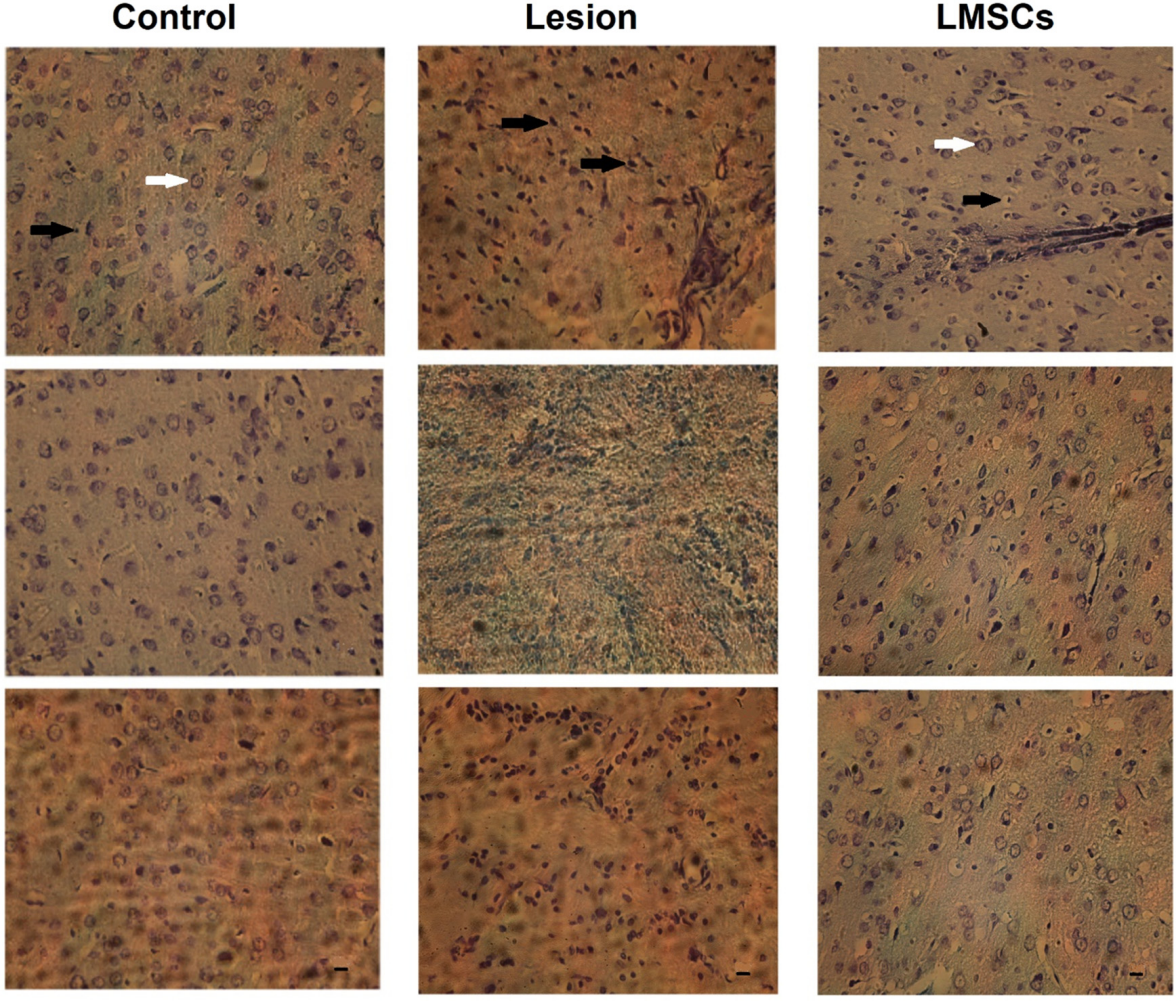
Neurons in the barrel cortex area, characterized by clear and visible nuclei and nucleoli, were considered viable and intact cells (white arrow). Depicted the degenerative cells (DC) had dark and pyknotic nuclei, dense and dark cytoplasm as (black arrow) in the lesion group increased compared to the control and LMSCs groups (magnification ×400).

**Figure 6:**
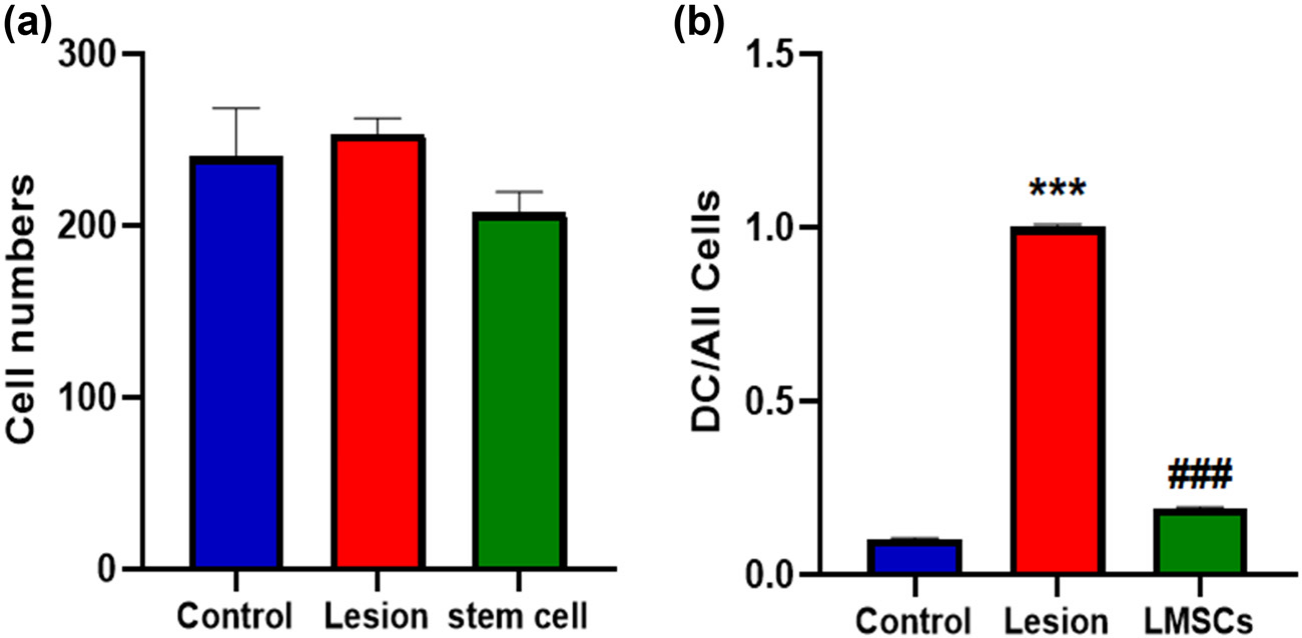
Effects of limbal stem cell transplantation on the number of cells (a) number of degenerated cells (b) of the barrel cortex. The data are presented as mean ± SEM. ***P < 0.001 vs Control, ^###^ *P* < 0.001 vs Lesion.

One-way ANOVA indicated a significant difference in the DC/All cell among groups in barrel cortex (F2, 6 = 6533, *P* = 0.000). After behavioral test histological evaluation was performed. Thirty-six, fields from the slides were carefully selected for examination, with matching among the groups. An optical microscope set to a magnification of ×400 (Figure 5) was used to evaluate of the samples. ANOVA followed by the Bonferroni post hoc analysis indicated that the number of neurons in the barrel cortex field remained unchanged across the experimental groups (Figure 6a). There was a significant increase in the DC/All cells ratio in the lesion group compared to the control group (*P* < 0.001). Additionally, both the control group and the LMSCs group exhibited a significant decrease in the DC/All cells ratio compared to the lesion group (*P* < 0.001) (Figure 6b).

## 4 Discussion

For the first time, this study examined the effects of direct transplantation of limbal stem cells into lesioned somatosensory cortex regions on behavioral performance and histology markers in rats. We found that directly transplanting LMSCs into lesioned somatosensory cortex regions could enhance functional recovery. Our evaluation involved an open-field test to assess the rats’ spontaneous spatial exploration ability. This choice was motivated by the knowledge that rodents primarily rely on their whiskers to explore their surroundings when navigating unfamiliar environments. Interestingly, our open-field experiment revealed a noticeable reduction in the distance covered by mice exploring the maze environment in the lesion group compared to the control group during the third week. In novel settings, rats often prefer maintaining whisker contact with walls or other vertical surfaces. This behavior is believed to help alleviate the inherent anxiety of traversing an open-field environment [25]. Our prior research shows the beneficial impact of rat dental pulp stem cell therapy on behavioral performance following brain injuries [13,26,27]. These findings provide valuable insights into the exploratory behavior of the rats and how it evolved over three weeks following the intervention. Research in the field indicates that MSC transplantation in brain-damaged areas reduces GFAP expression, enhances neural regeneration, and increases Olig2-positive cells, potentially improving functional outcomes in brain injury [13]. Limbal stem cells, also called corneal epithelial stem cells, are unipotent stem cells in the basal epithelial layer of the corneal limbus. They demarcate the boundary between the cornea and the sclera. Critical features of limbal stem cells include a slow turnover rate, high capacity for proliferation, clonogenicity, expression of specific stem cell markers, and the ability to regenerate the entire corneal epithelium [28]. Our findings revealed that texture discrimination abilities improved in animals undergoing limbal stem cell injections. These findings align with previous research, which has similarly hinted at the promise of LMSCs in ameliorating disruptions following cortical lesions [17].

Recent investigations have furnished compelling evidence of neurogenesis in humans following TBI. Neurogenesis is not random; it tends to concentrate around injured brain regions, where newly generated neurons migrate toward the boundary zone of the lesioned cerebral cortex [29]. Furthermore, in the consequences of TBI, the activation of endogenous neurogenesis initiates a process of spontaneous behavioral recovery [30]. The multifaceted roles of stem cells in tissue repair, neuroprotection, and trophic support position them as promising candidates for therapeutic intervention [31]. Notably, mesenchymal stem cell-based neural transplantation has emerged as a prospective treatment strategy for addressing TBI. Research shows mesenchymal stem cells can have anti-inflammatory properties by secreting cytokines like IL-10 and IL-6, while pro-inflammatory cytokines can trigger their anti-inflammatory effects. LMSC transplantation can improve neurological function post-stroke [32–34]. Furthermore, Calió et al. reported that MSC transplantation may shield brain tissue against oxidative stress and reduce apoptosis following stroke [35]. Our histological results showed that the number of neurons within the barrel cortex field remained consistent across all experimental groups. However, a notable finding emerged, revealing a substantial elevation in the ratio of DC/All cells within the lesion group compared to the control group. Furthermore, the control and LMSC transplantation groups indicated a marked reduction in the ratio of DC/All cells compared to the lesion group. Limbal stem cells, well-known for their regenerative capabilities within the ocular tissues, are investigated for their potential role in regenerative medicine [36]. These cells, typically located at the corneal-conjunctival boundary, have long been harnessed for their capacity to renew and restore the corneal epithelium, making them valuable in treating ocular injuries and diseases [37]. However, the appeal of limbal stem cells extends beyond their ocular domain. Tsai et al. highlighted the potential of these cells for neural differentiation, suggesting their benefits for neurological tissue regeneration [38]. While this avenue of research is in its infancy and necessitates further exploration, the multipotentiality of limbal stem cells underscores their significance in advancing regenerative medicine. It offers hope for addressing neurological injuries and disorders [38].

The timing of our stem cell transplantation, 72 h after cortical damage, was deliberate, as this is when neuronal apoptosis and necrosis are expected to be at their lowest levels [39]. In this study, we opted for allogeneic cell implantation directly into the lesioned region, given the more pronounced trophic effects associated with direct stem cell injection into the lesion site than other injection methods [40]. Soluble growth factors or cytokines may be present in the conditioned medium obtained from cultivated MSCs [41]. Investigating biological processes, such as shape, cell development and differentiation, proliferation, adhesion, migration, and secretion, occurs in this ‘niche’ milieu [41]. Neurite growth can be stimulated by neurotrophic agents [42]. Prior research has demonstrated that BMSCs and ADSCs can release various neurotrophic and growth factors, supporting neurite outgrowth and neuronal cell survival [43,44]. Similarly, a study found that L. conditioned medium enhanced cortical neuron outgrowth. This implies that LMSCs may release cytokines or neurotrophic factors, creating niches that aid in the proliferation of neurons. ELISA assay and the human growth factor antibody array map demonstrate that LMSCs release a number of essential neurotrophic factors, including VEGF, IGF-II, HGF, BDNF, GM-CSF, and VEGFR3 [17]. Decreases in VEGF and BDNF could explain hypoxia-treated LMSCs. In the current study, limitations were observed when utilizing tests like the Adhesive Removal test, Whisker test, and Von Frey Filament test due to potential confounding effects in the cohort studies. Subsequent research may gain advantages by integrating these tests to enhance the comprehensiveness of their findings. Applying multiple tests could impact treatment assessment and stress the animals. Hence, we opted for the NTD test to concurrently evaluate the open field test during the habituation day. The open field test can uncover motor deficits as well as exploration, aspects were monitored in this study. Additionally, a modified Von Frey filament test could assess pain in the barrel field, whereas our focus was on cognitive evaluation. However, additional research is needed to determine the underlying causes and specific molecular mechanisms. Compared to other MSC sources, LMSCs have a number of benefits, such as simple enrichment and quick growth rates, because of their propensity to stick to plastic. MSCs show enormous promise for treating human illnesses. Growth factors and cytokine release, in addition to direct MSC differentiation, may contribute to the therapeutic benefits of MSCs. L-MSCs have neurotrophic and neuroprotective properties. L-MSCs release paracrine factors that can accumulate in conditioned media during cell culture, promote neuronal growth, and mitigate the effects of cerebral ischemia and hypoxia-induced neuronal injury *in vivo* and *in vitro*, respectively. Potential roles for L-MSCs could offer different cell sources for studying and managing CNS illnesses [17]. Furthermore, our investigation aligns with previous studies that have highlighted the favorable influence of stem cell therapy on the behavioral performance of animals following brain injury. In our research, we detected a potential enhancement in the functioning of the contralateral barrel cortex [45]. However, it is essential to underscore the need for further investigations to comprehensively unveil the intricate mechanisms through which LMSCs exert their therapeutic effects.

## 5 Conclusion

In conclusion, our research has provided valuable insights into the potential therapeutic benefits of LMSCs transplantation in the context of cortical lesions. Through a series of behavioral tests, we have observed improvements in sensory function in rats that receiving LMSCs transplantation, particularly in their ability to discriminate between different textures.
